# Graphical presentation of diagnostic information

**DOI:** 10.1186/1471-2288-8-20

**Published:** 2008-04-11

**Authors:** Penny F Whiting, Jonathan AC Sterne, Marie E Westwood, Lucas M Bachmann, Roger Harbord, Matthias Egger, Jonathan J Deeks

**Affiliations:** 1 Department of Social Medicine, Canynge Hall, Whiteladies Road, Bristol, BS8 2PR, UK; 2 Centre for Reviews and Dissemination, University of York, YO10 5DD, UK; 3 Horten Centre, Zürich, Switzerland; 4 Department of Social and Preventive Medicine, University of Bern, Switzerland; 5 Medical Statistics Group/Diagnostic Research Group, Department of Public Health and Epidemiology, University of Birmingham, B15 2TT, UK

## Abstract

**Background:**

Graphical displays of results allow researchers to summarise and communicate the key findings of their study. Diagnostic information should be presented in an easily interpretable way, which conveys both test characteristics (diagnostic accuracy) and the potential for use in clinical practice (predictive value).

**Methods:**

We discuss the types of graphical display commonly encountered in primary diagnostic accuracy studies and systematic reviews of such studies, and systematically review the use of graphical displays in recent diagnostic primary studies and systematic reviews.

**Results:**

We identified 57 primary studies and 49 systematic reviews. Fifty-six percent of primary studies and 53% of systematic reviews used graphical displays to present results. Dot-plot or box-and- whisker plots were the most commonly used graph in primary studies and were included in 22 (39%) studies. ROC plots were the most common type of plot included in systematic reviews and were included in 22 (45%) reviews. One primary study and five systematic reviews included a probability-modifying plot.

**Conclusion:**

Graphical displays are currently underused in primary diagnostic accuracy studies and systematic reviews of such studies. Diagnostic accuracy studies need to include multiple types of graphic in order to provide both a detailed overview of the results (diagnostic accuracy) and to communicate information that can be used to inform clinical practice (predictive value). Work is required to improve graphical displays, to better communicate the utility of a test in clinical practice and the implications of test results for individual patients.

## Background

Readers of a research report evaluating a diagnostic test may wish to assess the test's characteristics (diagnostic accuracy) or evaluate the impact that its use has on diagnostic decisions (predictive value) for individual patients. Graphical displays of results of test accuracy studies allow researchers to summarise and communicate the key findings of their study. We discuss the types of graphical display commonly encountered in primary diagnostic accuracy studies and systematic reviews of such studies, and systematically review the use of graphical displays in recent diagnostic systematic reviews and primary studies. Table 1 defines the various measures of diagnostic accuracy used.

**Table 1:** Definitions of measures of diagnostic accuracy

		**Target condition**					
		Present	Absent				
**Test result**	**+**	**a**	**b**				
	**-**	**c**	**d**				
**Sensitivity**	*a/(a + c) - *Proportion of true positives that are correctly identified by the test [31]						

**Specificity**	*d/(b + d) - *Proportion of true negatives that are correctly identified by the test						

**Likelihood ratio (LR)**	Describes how may times a person with disease is more likely to receive a particular test result than a person without disease [32] The interpretation of likelihood ratios depends very much on clinical context. *Likelihood ratio for positive result (LR +) *= *[a/(a + c)]/[b/(b + d)]* = *sensitivity/(1 -specificity)* *Likelihood ratio for negative result (LR -) *= *[c/(a + c)]/[d/(b + d)]* = *(1 - sensitivity)/specificity*						

**Diagnostic odds ratio (DOR)**	Used as an overall (single indicator) measure of the diagnostic accuracy of a diagnostic test. It is calculated as the odds of positivity among diseased persons, divided by the odds of positivity among non-diseased. When a test provides no diagnostic evidence then the DOR is 1.0. [33] This measure has a number of limitations: by combining sensitivity and specificity into a single indicator the relative values of the two are lost i.e. the DOR can be the same for a very high sensitivity and low specificity as for very high specificity and low sensitivity [33] Further, tests that are effective for classifying persons as having or not having the target condition have DORs that whose magnitude is much greater (e.g. 100) than usually considered as indicating strong associations in epidemiological studies. [34] DOR = *[a/c]/ [b/d]* = *[sensitivity/(1 -specificity)]/[(1 - sensitivity)/specificity]* = *LR +ve/LR -ve* = *ad/bc*						

**Predictive value**	Positive predictive value: proportion of patients with positive test results who are correctly diagnosed *Positive predictive value (PPV) = a/ (a + b)* Negative predictive value: proportion of patients with negative test results who are correctly diagnosed *Negative predictive value (NPV) = d (c + d)* Predictive values depend on disease prevalence, the more common a disease is, the more likely it is that a positive test result is right and a negative result is wrong. [35]						

### Types of graphical display

#### Primary studies

**Figure 1:**
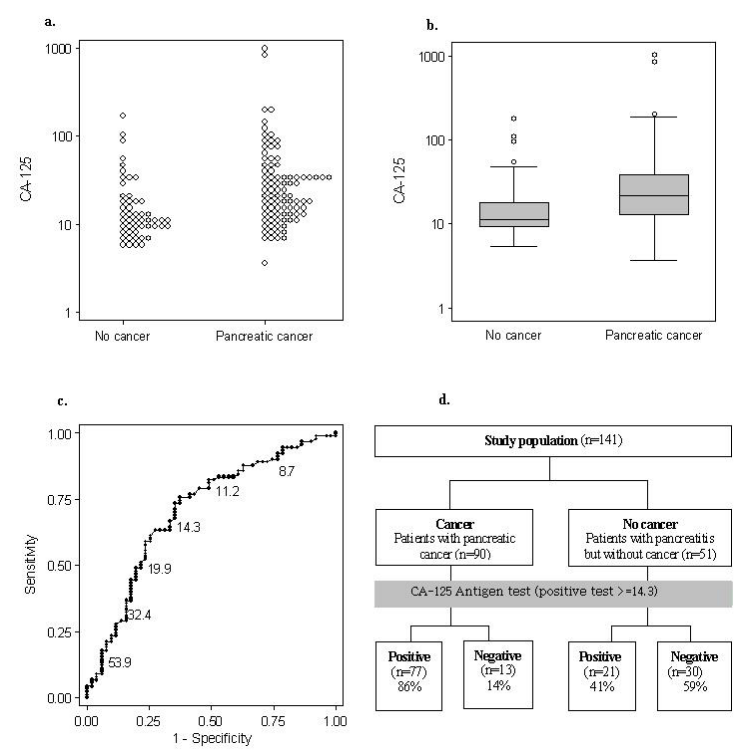
**Example graphical displays for primary study data**. a. Dot plot. b. Box-and-whisker plot. c. ROC Plot. d. Flow diagram.

Figure 1 illustrates four types of graphical display commonly used to present data on diagnostic accuracy for primary diagnostic accuracy studies. We used data from a study of the biochemical tumour marker CA-19-9 antigen to diagnose pancreatic cancer to construct these graphs [1].

#### Dot plots (Figure 1a) and Box-and-whisker plots (Figure 1b)

Dot plots are used for test results that take many values, and display the distribution of results in patients with and without the target condition. Box and whisker plots summarise these distributions: the central box covers the interquartile range with the median indicated by the line within the box. The whiskers extend either to the minimum and maximum values or to the most extreme values within 1.5 interquartile ranges of the quartiles, in which case more extreme values are plotted individually [2]. Sometimes an indication of the threshold used to define a positive test result is included, for example by adding a horizontal line or shading at the relevant point. Such plots can be used to clearly summarise a large volume of data, but are only able to display differences in the distribution of test values between patients with and without the target condition; they do not directly display the diagnostic performance of the test.

Although the CA-19-9 antigen test to diagnose pancreatic cancer (used to construct Figure 1) is an example of continuous data, it is also possible to construct similar graphs for categorical test results providing that the number of categories is reasonably large. Alternatively, for smaller numbers of categories, similar information can be conveyed using paired bar charts/histograms. Paired histograms show the distribution of test results in patients with the target condition above the x-axis and the distribution in patients without the target condition below the x-axis. These types of graphical display are less commonly used. It is not possible to construct any of these graphs for truly dichotomous test results. However, truly dichotomous tests rarely occur in practice. Examples of dichotomous tests include dipstick tests that change colour if the target condition is said to be present (although these are based on an underlying implicit threshold) or the presence/absence of certain clinical symptoms.

#### Receiver operating characteristic (ROC) plot (Figure 1c)

ROC plots show values of sensitivity and specificity at all of the possible thresholds that could be used to define a positive test result [3]. Typically, sensitivity (true positive rate) is plotted against 1-specificity (false positive rate): each point represents a different threshold in the same group of patients. Stepped lines are used for continuous test results while sloping lines are used for ordered categories. ROC curves may be derived directly from the observed sensitivity and specificity corresponding to different test thresholds, or by fitting curves based on parametric [4], semi-parametric [5,6], or non-parametric methods [7]. The *area under the ROC curve *(AUC) is a summary of diagnostic performance, and takes values between 0.5 and 1. The more accurate the test, the more closely the curve approaches the top left hand corner of the graph (AUC = 1). A test that provides no diagnostic information (AUC = 0.5) will produce a straight line from the bottom left to the top right. ROC curves may be restricted to a range of sensitivities or specificities of clinical interest.

ROC plots show how estimated sensitivity and specificity vary according to the threshold chosen, and can be used to identify suitable thresholds for clinical practice if the points on the curve are labelled with the corresponding threshold as in Figure 1c, which shows for example that the sensitivity and specificity corresponding to a threshold of 39.3 are 74% and 90%, respectively. Confidence intervals can be added to indicate the uncertainty in estimates of test performance at each point. ROC plots also allow comparison of the performance of several tests independently of choice of threshold, by plotting data sets for multiple tests in the same ROC space. However, they are thought to be difficult to interpret as they describe the characteristics of the test in a way which does not relate directly to its usefulness in clinical practice; research has shown that ROC plots are generally poorly understood by clinicians [8].

#### Flow charts (Figure 1d)

These depict the flow of patients through the study: for example how many patients were eligible, how many entered the study, how many of these had the target condition, and the numbers testing positive and negative. Such charts require categorisation of test results, for example as "positive" and "negative". Although flow charts do not directly present diagnostic accuracy data, addition of percentages to the test result boxes (as in Figure 1d) can be used to report test sensitivity (68/90 = 76%) and specificity (46/51 = 90%). Charts that first separate individuals according to test result before classification by disease status may similarly be used to depict positive and negative predictive values. The STARD (standards for reporting of diagnostic accuracy) statement, an initiative to improve the reporting of diagnostic test accuracy studies similar to the CONSORT statement for clinical trials, recommends the inclusion of a flow diagram in all reports of primary diagnostic accuracy studies [9]. This should illustrate the design of the study and provide information on the numbers of participants at each stage of the study as well as the results of the study. The example flow chart in Figure 1d is not a full STARD flow diagram as we do not have data on numbers of withdrawals or uninterpretable results from this study. It does, however, show the design (diagnostic case-control) and results of the study.

#### Systematic reviews

**Figure 2:**
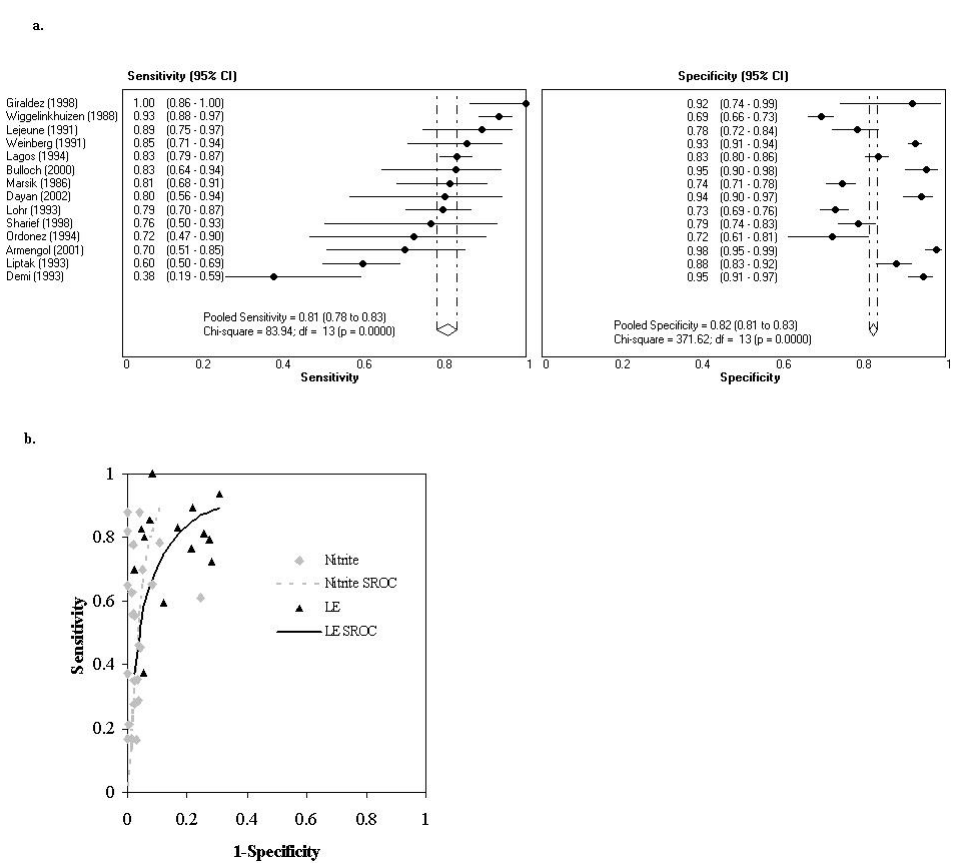
**Example graphs for systematic review data**. a. Paired forest plots of sensitivity and specificity for LE dipstick. b. ROC plot with SROC curves.

Figure 2 illustrates two graphical displays commonly used to present data on diagnostic accuracy in diagnostic systematic reviews. Data from a systematic review of dipstick tests for urinary nitrite and leukocyte esterase to diagnose urinary tract infections were used to construct these graphs [10].

#### Forest plots (Figure 2a)

Forest plots are commonly used to display results of meta-analysis. They display results from the individual studies together with, optionally, a summary (pooled) estimate. Point estimates are shown as dots or squares (sometimes sized according to precision or sample size) and confidence intervals as horizontal lines [11]. The pooled estimate is displayed as a diamond whose centre represents the estimate and tips the confidence interval.

For diagnostic accuracy studies, measures of test performance (sensitivity, specificity, predictive values, likelihood ratios or diagnostic odds ratio) are plotted on the horizontal axis. Diagnostic test performance is often described by pairs of summary statistics (e.g. sensitivity and specificity; positive and negative likelihood ratios), and these are depicted side-by-side. Between-study heterogeneity can readily be assessed by visual examination. Results may be sorted by one of a pair of test performance measures, usually that which is most important to the clinical application of the test. A disadvantage of paired forest plots is that they do not directly display the inverse association between the two measures that commonly results from variations in threshold between studies.

#### ROC plots and summary ROC (SROC) curves (Figure 2b)

**Figure 3:**
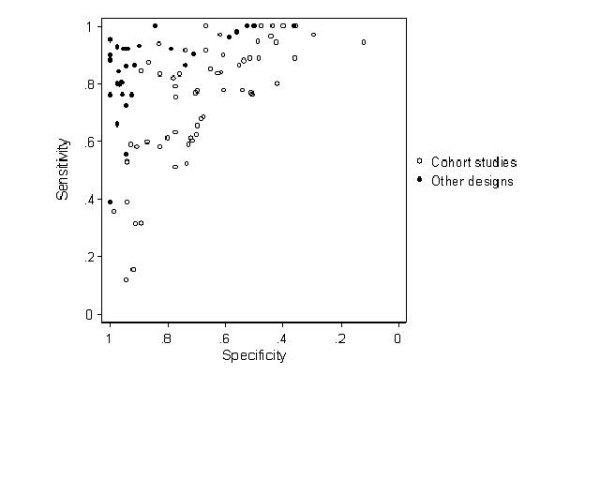
Sensitivity plotted against specificity, separately for cohort studies and for studies of other designs for MRI for diagnosis of multiple sclerosis.

ROC plots can be used to present the results of diagnostic systematic reviews, but differ from those used in primary studies as each point typically represents a separate study or data set within a study (individual studies may contribute more than one point). A summary ROC (SROC) curve can be estimated using one of several methods [12-15] and quantifies test accuracy and the association between sensitivity and specificity based on differences between studies. As with forest plots, ROC plots provide an overview of the results of all included studies. However, unless there are very few studies, it is not feasible to display confidence intervals as the plot would become cluttered. Results for several tests can be displayed on the same plot, facilitating test comparisons. It is also possible to display pooled estimates of sensitivity and specificity together with associated confidence intervals or prediction regions. ROC plots may also be used to investigate possible explanations for differences in estimates of accuracy between studies, for example those arising from differences in study quality. Figure 3 shows results for a recent review that we conducted on the accuracy of magnetic resonance imaging (MRI) for the diagnosis of multiple sclerosis (MS) [16]. By using different symbols to illustrate studies that did (diagnostic cohort studies) and did not (other study designs) include an appropriate patient spectrum we were able to show that studies that included an inappropriate patient spectrum grossly overestimated both sensitivity and specificity.

#### Other plots

Various other graphical methods have been developed to display the results of systematic reviews and meta-analyses [17,18]. Although not generally developed specifically for diagnostic test reviews these can be adapted to display the results of such reviews. Funnel plots [19] and Galbraith plots [20] are often used to assess evidence for publication bias or small study effects in systematic reviews of the effects of medical interventions assessed in randomized controlled trials. However, their application to systematic reviews of diagnostic test accuracy studies is problematic [20]. Diagnostic odds ratios are typically far from 1, and it has been shown that, for data of this type, sampling variation can lead to artefactual associations between log odds ratios and their standard errors [21]. It is therefore recommended that the effective sample size funnel plot be used in reviews of test accuracy studies [20].

#### Predictive value

**Figure 4:**
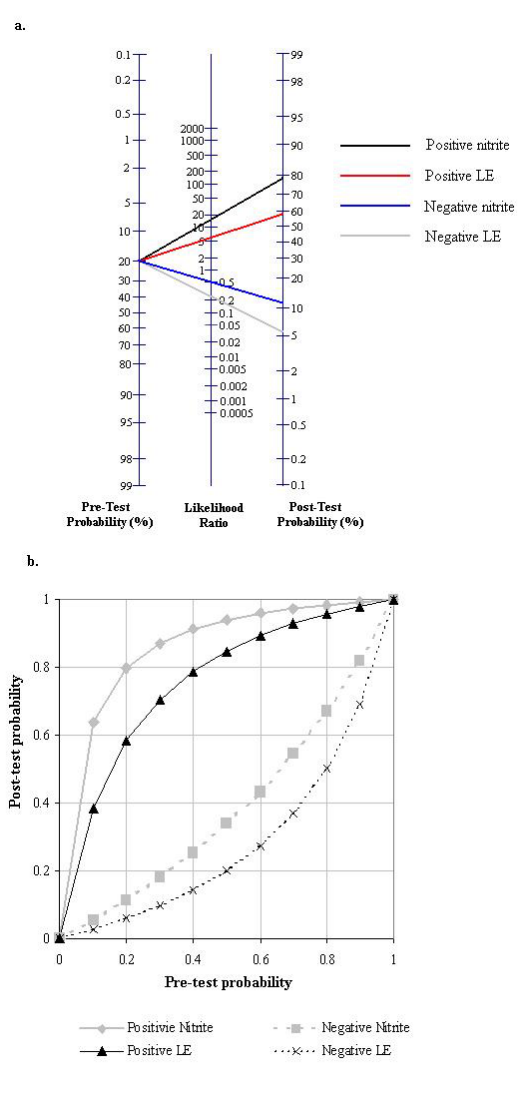
**Example graphs for interpreting diagnostic study result**. a. Likelihood ratio nomogram. b. Probability modifying plot.

A number of graphical displays aim to put results of diagnostic test evaluations into clinical context, based either on primary studies or systematic reviews. Two graphical displays commonly used for this purpose are the likelihood ratio nomogram (Figure 4a) and the probability-modifying plot (Figure 4b). Each allows the reader to estimate the post-test probability of the target condition in an individual patient, based on a selected pre-test probability. To use the likelihood ratio nomogram, the reader needs an estimate of the likelihood ratios for the test. He then draws a line through the appropriate likelihood ratio on the central axis, intersecting the selected pre-test probability, to derive the post-test probability of disease. The probability-modifying plot depicts separate curves for positive and negative test results. The reader draws a vertical line from the selected pre-test probability to the appropriate likelihood ratio line and then reads the post-test probability off the vertical scale. Both graph types are based on a single estimate of test accuracy (likelihood ratio), although it is possible to plot separate curves on the probability-modifying plot or lines on the nomogram to depict confidence intervals around the estimated likelihood ratios. Each assumes constant likelihood ratios across the range of pre-test probabilities. However, this assumption may be violated in practice [22], because populations in which the test is used may have different spectrums of disease to those in which estimates of test accuracy were derived.

## Use of graphical displays in the literature

### Methods

We systematically reviewed how graphical displays are currently incorporated in studies of test performance. We included primary diagnostic accuracy studies published in 2004, identified by hand searching 12 journals (Table 2), and diagnostic systematic reviews published in 2003, identified from DARE (Database of Abstracts of Reviews of Effects) [23]. Searches were conducted in 2005 and so these years were the most complete available years for searching (there is a delay in adding studies to DARE). Diagnostic accuracy studies were studies that provided data on the sensitivity and specificity of a diagnostic test and that focused on diagnostic (whether the patient had the condition of interest) rather than prognostic (disease severity/risk prediction) questions. Journals were selected to provide a mixture of the major general medical and specialty journals. We particularly aimed to select journals that clinicians read. We extracted data on the different graphical displays used to summarise information about test performance, defined as any graphical method of summarising data on diagnostic accuracy or the predictive value of a test (Table 1).

**Table 2:** Number of primary studies identified from the journals searched together with the number of studies from each journal that included graphical displays

*Journal*	*Number of studies*	*Number with graphs (%)*
Clinical Chemistry	25	18 (72)
American Journal of Obstetrics and Gynecology	1	0 (0)
Annals of Internal Medicine	6	3 (50)
BMJ	3	0 (0)
European Journal of Pediatrics	1	0 (0)
Gastroenterology	7	4 (57)
JAMA	5	2 (40)
British Journal of Radiology	1	1 (100)
Lancet	3	2 (67)
New England Journal of medicine	3	2 (67)
Thorax	2	0

We located 56 primary studies and 49 systematic reviews (Web Appendix). Fifty-seven percent of primary studies and 53% of systematic reviews used graphical displays to present results. In publications using graphics, the number of graphs per publication ranged from 1 to 51 (median 2, IQR 1 to 3 for primary studies and median 4, IQR 2 to 7 for systematic reviews). Table 3 summarises the categories of tests evaluated in the primary studies and systematic reviews. None of the tests evaluated in any of the primary studies were truly dichotomous: they all gave continuous or categorical results. Three of the eight systematic reviews that assessed clinical examination looked at whether a variety of signs or symptoms were present or absent: these can be considered as truly dichotomous tests. All other reviews evaluated continuous or categorical tests.

**Table 3:** Number of studies evaluating each category of tests in the primary studies and systematic reviews.

**Test category**	**Number of primary studies**	**Number of systematic reviews**
Clinical examination	4	8
Imaging	13	22
Laboratory	36	11
Questionnaires	0	3
Combination of different categories	3	4

### Primary studies

Dot-plots or box-and-whisker plots were the most commonly used graphic and were included in 22 (39%) studies. Generally the plots showed individual test results separately for patients with and without the target condition, with four including an indication of the threshold used to define a positive test result. Three studies included both a dot plot and a box-and-whisker plot on the same figure. Other variations included separate plots for different patient subgroups, different symbols to indicate different stages of disease, or separate plots for different tests. The majority of studies using these types of plots were of laboratory tests. An ROC curve was displayed in 15 (26%) studies. All of these plotted full ROC curves; only two provided any indication of the thresholds corresponding to one or more of the points. Thirteen studies included separate ROC curves for different tests, either on the same plot (10 studies) or on separate plots (3 studies). Five studies included separate ROC plots for different patient subgroups. Although all the primary studies were published in 2004, after the publication of the STARD guidelines, only one included a STARD flow diagram.

### Systematic reviews

ROC plots were included in 22 (45%) reviews. Twenty showed individual study estimates of sensitivity and specificity, 14 fitted SROC curves, and two displayed a summary point. One study, which did not fit an SROC curve, added a box and whisker plot to each axis to show the distributions of sensitivity and specificity. One study plotted only summary estimates of sensitivity and specificity in ROC space, with no SROC curves. Some reviews included separate plots for different tests, for different patient subgroups, or for different thresholds used to define a positive test result.

Ten reviews (20%) used forest plots to display individual study results. One study provided a plot of diagnostic odds ratios, while all others displayed paired plots of sensitivity and specificity (8 reviews), positive and negative likelihood ratios (3 reviews), or positive and negative predictive values (1 review). Several studies displayed more than one set of forest plots, including plots for more than one summary measure, for different stages of diagnosis, different test thresholds or for different tests. One study included a forest plot of summary data only, showing how pooled estimates of positive and negative likelihood ratios varied for different patient subgroups.

### Predictive value

None of the studies included a likelihood ratio nomogram. One primary study and five systematic reviews included a probability-modifying plot.

## Discussion

Research in the area of cognitive psychology suggests that sensitivity and specificity are generally poorly understood by doctors [8,24] and are often confused with predictive values [8,25,26]. Doctors tend to overestimate the impact of a positive test result on the probability of disease [27,28] and this overestimation increases with decreasing pre-test probabilities of disease [29]. This research suggests that the most informative measures for doctors may be estimates of the post-test probability of disease (predictive value), which can be presented as a range corresponding to different pre-test probabilities. However, graphical displays that facilitate the derivation of post-test probabilities, such as likelihood ratio nomograms, are usually based on summary estimates of test characteristics (positive and negative likelihood ratios) without allowing for the precision of the estimate, or its applicability to a given population. Use of summary estimates in this way is questionable in the context of reviews of diagnostic accuracy studies, which typically find substantial between-study heterogeneity [30]. It is particularly problematic if the summary estimate is the only information conveyed in a graphic and the graphic is taken as the key message of the paper.

The inclusion of some form of graphical presentation of test accuracy data has a number of advantages compared to not using such displays. It allows fuller reporting of results, for example (S)ROC plots can display results for multiple thresholds whereas reporting test accuracy results in a text or table generally requires the selection of one or more thresholds. In addition, (S)ROC plots depict the trade-off between sensitivity and specificity at different thresholds. Use of such displays also have the advantage of presenting all of the results of a primary study or systematic review without the need for selected analyses, which may be biased depending on the analyses selected. The inclusion of graphical displays, such as SROC plots or forest plots, in systematic reviews of test accuracy studies allows a visual assessment of heterogeneity between studies by showing the results from each individual study included in the review. There is also a suggestion that graphical displays may be easier to interpret than text or tabular summaries of the same data.

Diagnostic accuracy studies will usually need to include more than one graphic in order both to provide a detailed description of results (diagnostic accuracy) and to communicate appropriate summary measures that can be used to inform clinical practice (predictive value); the more detailed graphic provides context for the interpretation of summary measures. Further work is required to improve on existing graphical displays. The starting point for this should be further evaluation of the types of graphical display most helpful to assessing the utility of a test in clinical practice and the implications of test results for individual patients.

We hope that this paper will contribute to an increase in the use and quality of graphical displays in diagnostic accuracy studies and systematic reviews of these studies. To achieve this, journal guidelines and the STARD statement need to encourage the use of graphs in reports of test accuracy. Currently, journal guidelines say very little about this issue. A brief review of the instructions for authors from a selection of leading medical journals (*Annals of Internal Medicine*, *BMJ*, *Clinical Chemistry*, *JAMA*, *Lancet*, *New England Journal of Medicine*) found that these only provide formatting guidelines rather than discussing when and what type of graphical displays should be used, although all except the *New England Journal of Medicine *recommend that the STARD guidelines be followed and include references to the STARD flow diagram. STARD itself does not comment on how graphical displays should be used to convey results of test accuracy studies other than to recommend the inclusion of a flow diagram and to provide an illustration of a dot-plot as a suggestion for how individual study results may be displayed. Guidelines on the type of graphical displays that should be included in reports of test accuracy studies could be considered when STARD is next updated, and should be considered by journals in their instructions for authors.

## Conclusion

Our review suggests that graphical displays are currently underused in primary diagnostic accuracy studies and systematic reviews of such studies. Graphical displays of diagnostic accuracy data should provide an easily interpretable and accurate representation of study results, conveying both diagnostic accuracy and predictive value. This is not usually possible in a single graphic: the type of information presented in the most commonly used graphs does not directly allow clinicians to assess the implications of test results for an individual patient.

## Competing interests

The author(s) declare that they have no competing interests.

## Authors' contributions

All authors contributed to the design of the study and read and approved the final manuscript. PFW and MEW identified relevant studies and extracted data from included studies. PFW carried out the analysis and drafted the manuscript with help from JD and RH.

## Web Appendix: Studies included in the review

### a. Primary studies

1. Arvanitakis M, Delhaye M, De Maertelaere V, Bali M, Winant C, Coppens E, et al. Computed tomography and magnetic resonance imaging in the assessment of acute pancreatitis. Gastroenterology 2004;126:715–723.

2. Baldas V, Tommasini A, Santon D, Not T, Gerarduzzi T, Clarich G, et al. Testing for Anti-Human Transglutaminase Antibodies in Saliva Is Not Useful for Diagnosis of Celiac Disease. Clin Chem 2004;50:216–219.

3. Banks E, Reeves G, Beral V, Bull D, Crossley B, Simmonds M, et al. Influence of personal characteristics of individual women on sensitivity and specificity of mammography in the Million Women Study: cohort study. BMJ 2004;329:477.

4. Baschat AA, Guclu S, Kush ML, Gembruch U, Weiner CP, Harman CR. Venous Doppler in the prediction of acid-base status of growth-restricted fetuses with elevated placental blood flow resistance. Am J Obstet Gynecol 2004;191:277–284.

5. Biel SS, Nitsche A, Kurth A, Siegert W, Ozel M, Gelderblom HR. Detection of Human Polyomaviruses in Urine from Bone Marrow Transplant Patients: Comparison of Electron Microscopy with PCR. Clin Chem 2004;50:306–312.

6. Bluemke DA, Gatsonis CA, Chen MH, DeAngelis GA, DeBruhl N, Harms S, et al. Magnetic Resonance Imaging of the Breast Prior to Biopsy. JAMA 2004;292:2735–2742.

7. Brugge WR, Lewandrowski K, Lee-Lewandrowski E, Centeno BA, Szydlo T, Regan S, et al. Diagnosis of pancreatic cystic neoplasms: a report of the cooperative pancreatic cyst study. Gastroenterology 2004;126:1330–1336.

8. Bulterys M, Jamieson DJ, O'Sullivan MJ, Cohen MH, Maupin R, Nesheim S, et al. Rapid HIV-1 Testing During Labor: A Multicenter Study. JAMA 2004;292:219–223.

9. Carnevale V, Dionisi S, Nofroni I, Romagnoli E, Paglia F, De Geronimo S, et al. Potential Clinical Utility of a New IRMA for Parathyroid Hormone in Postmenopausal Patients with Primary Hyperparathyroidism. Clin Chem 2004;50:626–631.

10. Chye SM, Lin SR, Chen YL, Chung LY, Yen CM. Immuno-PCR for Detection of Antigen to Angiostrongylus cantonensis Circulating Fifth-Stage Worms. Clin Chem 2004;50:51–57.

11. Cotton PB, Durkalski VL, Pineau BC, Palesch YY, Mauldin PD, Hoffman B, et al. Computed Tomographic Colonography (Virtual Colonoscopy): A Multicenter Comparison With Standard Colonoscopy for Detection of Colorectal Neoplasia. JAMA 2004;291:1713–1719.

12. DeWitt J, Devereaux B, Chriswell M, McGreevy K, Howard T, Imperiale TF, et al. Comparison of Endoscopic Ultrasonography and Multidetector Computed Tomography for Detecting and Staging Pancreatic Cancer. Ann Intern Med 2004;141:753–763.

13. Esteban A, Fernandez-Segoviano P, Frutos-Vivar F, Aramburu JA, Najera L, Ferguson ND, et al. Comparison of Clinical Criteria for the Acute Respiratory Distress Syndrome with Autopsy Findings. Ann Intern Med 2004;141:440–445.

14. Foxman EF, Jarolim P. Use of the Fetal Fibronectin Test in Decisions to Admit to Hospital for Preterm Labor. Clin Chem 2004;50:663–665.

15. Gibot S, Cravoisy A, Levy B, Bene MC, Faure G, Bollaert PE. Soluble Triggering Receptor Expressed on Myeloid Cells and the Diagnosis of Pneumonia. NEJM 2004;350:451–458.

16. Greenough A, Thomas M, Dimitriou G, Williams O, Johnson A, Limb E, et al. Prediction of outcome from the chest radiograph appearance on day 7 of very prematurely born infants. Eur J Pediatr 2004;163:14–18.

17. Grenache DG, Hankins K, Parvin CA, Gronowski AM. Cervicovaginal Interleukin-6, Tumor Necrosis Factor-, and Interleukin-2 Receptor as Markers of Preterm Delivery. Clin Chem 2004;50:1839–1842.

18. Hammerer-Lercher A, Ludwig W, Falkensammer G, Muller S, Neubauer E, Puschendorf B, et al. Natriuretic Peptides as Markers of Mild Forms of Left Ventricular Dysfunction: Effects of Assays on Diagnostic Performance of Markers. Clin Chem 2004;50:1174–1183.

19. Hattori H, Kujiraoka T, Egashira T, Saito E, Fujioka T, Takahashi S, et al. Association of Coronary Heart Disease with Pre-[beta]-HDL Concentrations in Japanese Men. Clin Chem 2004;50:589–595.

20. Herget-Rosenthal S, Poppen D, Husing J, Marggraf G, Pietruck F, Jakob HG, et al. Prognostic Value of Tubular Proteinuria and Enzymuria in Nonoliguric Acute Tubular Necrosis. Clin Chem 2004;50:552–558.

21. Hetzel M, Hetzel J, Arslandemir C, Nussle K, Schirrmeister H. Reliability of symptoms to determine use of bone scans to identify bone metastases in lung cancer: prospective study. BMJ 2004;328:1051–1052.

22. Hift RJ, Davidson BP, van der Hooft C, Meissner DM, Meissner PN. Plasma Fluorescence Scanning and Fecal Porphyrin Analysis for the Diagnosis of Variegate Porphyria: Precise Determination of Sensitivity and Specificity with Detection of Protoporphyrinogen Oxidase Mutations as a Reference Standard. Clin Chem 2004;50:915–923.

23. Hong KM, Najjar H, Hawley M, Press RD. Quantitative Real-Time PCR with Automated Sample Preparation for Diagnosis and Monitoring of Cytomegalovirus Infection in Bone Marrow Transplant Patients. Clin Chem 2004;50:846–856.

24. Imperiale TF, Ransohoff DF, Itzkowitz SH, Turnbull BA, Ross ME, the Colorectal Cancer Study Group. Fecal DNA versus Fecal Occult Blood for Colorectal-Cancer Screening in an Average-Risk Population. NEJM 2004;351:2704–2714.

25. Jung K, Reiche J, Boehme A, Stephan C, Loening SA, Schnorr D, et al. Analysis of Subforms of Free Prostate-Specific Antigen in Serum by Two-Dimensional Gel Electrophoresis: Potential to Improve Diagnosis of Prostate Cancer. Clin Chem 2004;50:2292–2301.

26. Kageyama S, Isono T, Iwaki H, Wakabayashi Y, Okada Y, Kontani K, et al. Identification by Proteomic Analysis of Calreticulin as a Marker for Bladder Cancer and Evaluation of the Diagnostic Accuracy of Its Detection in Urine. Clin Chem 2004;50:857–866.

27. Kiesslich R, Burg J, Vieth M, Gnaendiger J, Enders M, Delaney P, et al. Confocal laser endoscopy for diagnosing intraepithelial neoplasias and colorectal cancer in vivo. Gastroenterology 2004;127:706–713.

28. Kramer H, van Putten JWG, Post WJ, van Dullemen HM, Bongaerts AHH, Pruim J, et al. Oesophageal endoscopic ultrasound with fine needle aspiration improves and simplifies the staging of lung cancer. Thorax 2004;59:596–601.

29. Kriege M, Brekelmans CTM, Boetes C, Besnard PE, Zonderland HM, Obdeijn IM, et al. Efficacy of MRI and Mammography for Breast-Cancer Screening in Women with a Familial or Genetic Predisposition. NEJM 2004;351:427–437.

30. Lacey JM, Minutti CZ, Magera MJ, Tauscher AL, Casetta B, McCann M, et al. Improved Specificity of Newborn Screening for Congenital Adrenal Hyperplasia by Second-Tier Steroid Profiling Using Tandem Mass Spectrometry. Clin Chem 2004;50:621–625.

31. Lennon PV, Wingerchuk DM, Kryzer TJ, Pittock SJ, Lucchinetti CF, Fujihara K, et al. A serum autoantibody marker of neuromyelitis optica: distinction from multiple sclerosis. Lancet 2004;364:2106–2112.

32. Leung Sf, Tam JS, Chan ATC, Zee B, Chan LYS, Huang DP, et al. Improved Accuracy of Detection of Nasopharyngeal Carcinoma by Combined Application of Circulating Epstein-Barr Virus DNA and Anti-Epstein-Barr Viral Capsid Antigen IgA Antibody. Clin Chem 2004;50:339–345.

33. Leung GM, Rainer TH, Lau FL, Wong IOL, Tong A, Wong TW, et al. A Clinical Prediction Rule for Diagnosing Severe Acute Respiratory Syndrome in the Emergency Department. Ann Intern Med 2004;141:333–342.

34. Liebeschuetz S, Bamber S, Ewer K, Deeks J, Pathan AA, Lalvani A. Diagnosis of tuberculosis in South African children with a T-cell-based assay: a prospective cohort study. Lancet 2004;364:2196–2203.

35. Llorente MJ, Sebastián M, Fernández-Aceñero MJ, Prieto G, Villanueva S. IgA Antibodies against Tissue Transglutaminase in the Diagnosis of Celiac Disease: Concordance with Intestinal Biopsy in Children and Adults. Clin Chem 2004;50:451–453.

36. McLean RG, Carolan M, Bui C, Arvela O, Ford JC, Chew M, et al. Comparison of new clinical and scintigraphic algorithms for the diagnosis of pulmonary embolism. Br J Radiol 2004;77:372–376.

37. Miglioretti DL, Rutter CM, Geller BM, Cutter G, Barlow WE, Rosenberg R, et al. Effect of Breast Augmentation on the Accuracy of Mammography and Cancer Characteristics. JAMA 2004;291:442–450.

38. Mikolajczyk SD, Catalona WJ, Evans CL, Linton HJ, Millar LS, Marker KM, et al. Proenzyme Forms of Prostate-Specific Antigen in Serum Improve the Detection of Prostate Cancer. Clin Chem 2004;50:1017–1025.

39. Minguez M, Herreros B, Sanchiz V, Hernandez V, Almela P, AnonAnon R, et al. Predictive value of the balloon expulsion test for excluding the diagnosis of pelvic floor dyssynergia in constipation. Gastroenterology 2004;126:57–62.

40. Palomaki GE, Neveux LM, Knight GJ, Haddow JE, Pandian R. Maternal Serum Invasive Trophoblast Antigen (Hyperglycosylated hCG) as a Screening Marker for Down Syndrome during the Second Trimester. Clin Chem 2004;50:1804–1808.

41. Palomaki GE, Knight GJ, Roberson MM, Cunningham GC, Lee JE, Strom CM, et al. Invasive Trophoblast Antigen (Hyperglycosylated Human Chorionic Gonadotropin) in Second-Trimester Maternal Urine as a Marker for Down Syndrome: Preliminary Results of an Observational Study on Fresh Samples. Clin Chem 2004;50:182–189.

42. Papadopoulos MC, Abel PM, Agranoff D, Stich A, Tarelli E, Bell PBA, et al. A novel and accurate diagnostic test for human African trypanosomiasis. Lancet 2004;363:1358–1363.

43. Parsi MA, Shen B, Achkar JP, Remzi FF, Goldblum JR, Boone J, et al. Fecal lactoferrin for diagnosis of symptomatic patients with ileal pouch-anal anastomosis. Gastroenterology 2004;126:1280–1286.

44. Raad I, Hanna HA, Alakech B, Chatzinikolaou I, Johnson MM, Tarrand J. Differential Time to Positivity: A Useful Method for Diagnosing Catheter-Related Bloodstream Infections. Ann Intern Med 2004;140:18–25.

45. Rathbun SW, Whitsett TL, Raskob GE. Negative D-dimer Result To Exclude Recurrent Deep Venous Thrombosis: A Management Trial. Ann Intern Med 2004;141:839–845.

46. Rietveld RP, Riet Gt, Bindels PJE, Sloos JH, van Weert HCPM. Predicting bacterial cause in infectious conjunctivitis: cohort study on informativeness of combinations of signs and symptoms. BMJ 2004;329:206–210.

47. Rosenberg WMC, Voelker M, Thiel R, Becka M, Burt A, Schuppan D, et al. Serum markers detect the presence of liver fibrosis: A cohort study. Gastroenterology 2004;127:1704–1713.

48. Schwertz E, Kahlenberg F, Sack U, Richter T, Stern M, Conrad K, et al. Serologic Assay Based on Gliadin-Related Nonapeptides as a Highly Sensitive and Specific Diagnostic Aid in Celiac Disease. Clin Chem 2004;50:2370–2375.

49. van Gelder RE, Nio CY, Florie J, Bartelsman JF, Snel P, de Jager SW, et al. Computed tomographic colonography compared with colonoscopy in patients at increased risk for colorectal cancer. Gastroenterology 2004;127:41–48.

50. Van Meensel B, Hiele M, Hoffman I, Vermeire S, Rutgeerts P, Geboes K, et al. Diagnostic Accuracy of Ten Second-Generation (Human) Tissue Transglutaminase Antibody Assays in Celiac Disease. Clin Chem 2004;50:2125–2135.

51. Vasbinder GB, Nelemans PJ, Kessels AGH, Kroon AA, Maki JH, Leiner T, et al. Accuracy of Computed Tomographic Angiography and Magnetic Resonance Angiography for Diagnosing Renal Artery Stenosis. Ann Intern Med 2004;141:674–682.

52. Vlahou A, Giannopoulos A, Gregory BW, Manousakas T, Kondylis FI, Wilson LL, et al. Protein Profiling in Urine for the Diagnosis of Bladder Cancer. Clin Chem 2004;50:1438–1441.

53. Warner E, Plewes DB, Hill KA, Causer PA, Zubovits JT, Jong RA, et al. Surveillance of BRCA1 and BRCA2 Mutation Carriers With Magnetic Resonance Imaging, Ultrasound, Mammography, and Clinical Breast Examination. JAMA 2004;292:1317–1325.

54. Wasmuth J-C, Grün B, Terjung B, Homrighausen, Spengler A, Spengler U. ROC Analysis Comparison of Three Assays for the Detection of Antibodies against Double-Stranded DNA in Serum for the Diagnosis of Systemic Lupus Erythematosus. Clin Chem 2004;50:2169–2171.

55. Wildi SM, Judson MA, Fraig M, Fickling WE, Schmulewitz N, Varadarajulu S, et al. Is endosonography guided fine needle aspiration (EUS-FNA) for sarcoidosis as good as we think? Thorax 2004;59:794–799.

56. Zehentner BK, Persing DH, Deme A, Toure P, Hawes SE, Brooks L, et al. Mammaglobin as a Novel Breast Cancer Biomarker: Multigene Reverse Transcription-PCR Assay and Sandwich ELISA. Clin Chem 2004;50:2069–2076.

### b. Systematic reviews

1. Arbyn M, Schenck U, Ellison E, Hanselaar A. Metaanalysis of the accuracy of rapid prescreening relative to full screening of pap smears. Cancer Cytopathology 2003;99(1):9–16.

2. Austin MP, Lumley J. Antenatal screening for postnatal depression: a systematic review. Acta Psychiatr Scand 2003;107:10–17.

3. Babu AN, Kymes SM, Fryer SM. Eponyms and the diagnosis of aortic regurgitation: what says the evidence. Ann Intern Med 2003;138:736–742.

4. Bachmann LM, Kolb E, Koller MT, Steurer J, ter RG. Accuracy of Ottawa ankle rules to exclude fractures of the ankle and mid-foot: systematic review. BMJ 2003;326:417–419.

5. Bastian LA, Smith CM, Nanda K. Is this woman perimenopausal? JAMA 2003;289:895–902.

6. Bipat S, Glas AS, van d, V, Zwinderman AH, Bossuyt PM. Computed tomography and magnetic resonance imaging in staging of uterine cervical carcinoma: a systematic review. Gynecol Oncol 2003;91:59–66.

7. Boustani, M., Peterson, B., Hanson, L., Harris, R., and Lohr, K. N. Screening for dementia. 2003.

8. Cardarelli R, Lumicao TG. B-type natriuretic peptide: a review of its diagnostic, prognostic, and therapeutic monitoring value in heart failure for primary care physicians. J Am Board Fam Pract 2003;16:327–333.

9. Carnero-Pardo C. Systematic review of the value of positron emission tomography in the diagnosis of Alzheimer's disease. Rev Neurol 2003;37:860–870.

10. Chunilal SD, Eikelboom JW, Attia J, Miniati M, Panju AA, Simel DL. Does this patient have pulmonary embolism? JAMA 2003;290:2849–2858.

11. Dinnes J, Loveman E, McIntyre L, Waugh N. The effectiveness of diagnostic tests for the assessment of shoulder pain due to soft tissue disorders: a systematic review. Health Technol Assess 2003;7:1–178.

12. Farquhar C, Ekeroma A, Furness S, Arroll B. A systematic review of transvaginal ultrasonography, sonohysterography and hysteroscopy for the investigation of abnormal uterine bleeding in premenopausal women. Acta Obstet Gynecol Scand 2003;82:493–504.

13. Framarin, A. First-trimester prenatal screening for Down syndrome and other aneuploidies. Montreal, PQ, Canada. Agence d'Evaluation des Technologies et des Modes d'Intervention en Sante (AETMIS) 2003: 81.

14. Gilbert DL, Sethuraman G, Kotagal U, Buncher CR. Meta-analysis of EEG test performance shows wide variation among studies. Neurology 2003;60:564–570.

15. Glas AS, Roos D, Deutekom M, Zwinderman AH, Bossuyt PM, Kurth KH. Tumor markers in the diagnosis of primary bladder cancer: a systematic review. J Urol 2003;169:1975–1982.

16. Goerres GW, Mosna-Firlejczyk K, Steurer J, von Schulthess GK. Assessment of clinical utility of F-18-FDG PET in patients with head and neck cancer: a probability analysis. Eur J Nucl Med Mol Imaging 2003;30:562–571.

17. Goto M, Noguchi Y, Koyama H, Hira K, Shimbo T, Fukui T. Diagnostic value of adenosine deaminase in tuberculous pleural effusion: a meta-analysis. Ann Clin Biochem 2003;40:374–381.

18. Gould MK, Kuschner WG, Rydzak CE, Maclean CC, Demas AN, Chan JK, et al. Test performance of positron emission tomography and computed tomography for mediastinal staging in patients with non-small-cell lung cancer. Ann Intern Med 2003;139:879–892.

19. Grady, D., McDonald, K., Bischoff, K., Cabou, A., Chaput, L., Hoerster, K., Shahpar, C., Walsh, J., Sorrough, G., and Won, G. Results of systematic review of research on diagnosis and treatment of coronary heart disease in women. Rockville, MD, USA, Agency for Healthcare Research and Quality. 2003; 268

20. Gupta S, Bent S, Kohlwes J. Test characteristics of alpha-fetoprotein for detecting hepatocellular carcinoma in patients with hepatitis C. Ann Intern Med 2003;139:46–50.

21. Heffner JE, Highland K, Brown LK. A meta-analysis derivation of continuous likelihood ratios for diagnosing pleural fluid exudates. Am J Respir Crit Care Med 2003;167:1591–1599.

22. Hollingworth W, Nathens AB, Kanne JP, Crandall ML, Crummy TA, Wang MC, et al. The diagnostic accuracy of computed tomography angiography for traumatic or atherosclerotic lesions of the carotid and vertebral arteries: a systematic review. Eur J Radiol 2003;48:88–102.

23. Honest H, Bachmann LM, Coomarasamy A, Gupta JK, Kleijnen J, Khan KS. Accuracy of cervical transvaginal sonography in predicting preterm birth: a systematic review. Ultrasound Obstet Gynecol 2003;22:305–322.

24. Ioannidis JP, Lau J. F-18-FDG PET for the diagnosis and grading of soft-tissue sarcoma: a meta-analysis. J Nucl Med 2003;44:717–724.

25. Jackson JL, O'Malley PG, Kroenke K. Evaluation of acute knee pain in primary care. Ann Intern Med 2003;139:575–588.

26. Johnston R, V, Burrows E, Raulli A. Assessment of diagnostic tests to inform policy decisions-visual electrodiagnosis. Int J Technol Assess Health Care 2003;19:373–383.

27. Liberman M, Sampalis F, Mulder DS, Sampalis JS. Breast cancer diagnosis by scintimammography: a meta-analysis and review of the literature. Breast Cancer Res Treat 2003;80:115–126.

28. Makrydimas G, Sotiriadis A, Ioannidis JP. Screening performance of first-trimester nuchal translucency for major cardiac defects: a meta-analysis. Am J Obstet Gynecol 2003;189:1330–1335.

29. Neumayer L, Kennedy A. Imaging in appendicitis: a review with special emphasis on the treatment of women. Obstet Gynecol 2003;102:1404–1409.

30. Olaniyan OB. Validity of colposcopy in the diagnosis of early cervical neoplasia: a review. Afr J Reprod Health 2002;6:59–69.

31. Pai M, Flores LL, Pai N, Hubbard A, Riley LW, Colford JM. Diagnostic accuracy of nucleic acid amplification tests for tuberculous meningitis: a systematic review and meta-analysis. Lancet Infect Dis 2003;3:633–643.

32. Pasternack I, I, Malmivaara A, Tervahartiala P, Forsberg H, Vehmas T. Magnetic resonance imaging findings in respect to carpal tunnel syndrome. Scand J Work Environ Health 2003;29:189–196.

33. Pastor-Gomez J, Pulido-Rivas P, De Sola RG. Review of the literature on the value of magnetoencephalography in epilepsy. Rev Neurol 2003;37:951–996.

34. Patton LL. The effectiveness of community-based visual screening and utility of adjunctive diagnostic aids in the early detection of oral cancer. Eur J Cancer 2003;39:708–723.

35. Pirozzo S, Papinczak T, Glasziou P. Whispered voice test for screening for hearing impairment in adults and children: systematic review. BMJ 2003;327:967–970.

36. Rietveld RP, Van Weert HC, ter RG, Bindels PJ. Diagnostic impact of signs and symptoms in acute infectious conjunctivitis: systematic literature search. BMJ 2003;327:789.

37. Riley RD, Burchill SA, Abrams KR, Heney D, Lambert PC, Jones DR, et al. A systematic review and evaluation of tumour markers in paediatric oncology: Ewing's sarcoma and neuroblastoma. Health Technol Assess 2003;7:1–162.

38. Romagnuolo J, Bardou M, Rahme E, Joseph L, Reinhold C, Barkun AN. Magnetic resonance cholangiopancreatography: a meta-analysis of test performance in suspected biliary disease. Ann Intern Med 2003;139:547–557.

39. Rosado B, Menzies S, Harbauer A, Pehamberger H, Wolff K, Binder M, et al. Accuracy of computer diagnosis of melanoma: a quantitative meta-analysis. Arch Dermatol 2003;139:361–367.

40. Rothman R, Owens T, Simel DL. Does this child have acute otitis media? JAMA 2003;290:1633–1640.

41. Scholten RJ, Opstelten W, van der Plas CG, Bijl D, Deville WL. Accuracy of physical diagnostic tests for assessing ruptures of the anterior cruciate ligament: a meta-analysis. J Fam Pract 2003;52:689–694.

42. Sotiriadis A, Makrydimas G, Ioannidis JP. Diagnostic performance of intracardiac echogenic foci for Down syndrome: a meta-analysis. Obstet Gynecol 2003;101:1009–1016.

43. Takata GS, Chan LS, Morphew T, Mangione-Smith R, Morton SC. Evidence assessment of the accuracy of methods of diagnosing middle ear effusion in children with otitis media with effusion. Pediatrics 2003;112:1379–1387.

44. Toloza EM, Harpole L, McCrory DC. Noninvasive staging of non-small cell lung cancer: a review of the current evidence. Chest 2003;123:137S-146S.

45. Toloza EM, Harpole L, Detterbeck F, McCrory DC. Invasive staging of non-small cell lung cancer: a review of the current evidence. Chest 2003;123:157S-166S.

46. Trowbridge RL, Rutkowski NK, Shojania KG. Does this patient have acute cholecystitis? JAMA 2003;289:80–86.

47. van Gelder JM. Computed tomographic angiography for detecting cerebral aneurysms: Implications of aneurysm size distribution for the sensitivity, specificity, and likelihood ratios. Neurosurgery 2003;53:597–605.

48. Watson LC, Pignone MP. Screening accuracy for late-life depression in primary care: a systematic review. J Fam Pract 2003;52:956–964.

49. Watson EJ, Templeton A, Russell I, Paavonen J, Mardh PA, Stary A. The accuracy and efficacy of screening tests for Chlamydia trachomatis: a systematic review. J Med Microbiol 2002;51:1021–1031.

## Pre-publication history

The pre-publication history for this paper can be accessed here:


